# On the Manner in Which Phosphate of Lime Enters Organized Beings

**Published:** 1847-03

**Authors:** M. Dumas


*On the Manner in which Phosphate of Lime enters organized Beings,* by M. Dumas.—The phosphate of lime is insoluble in water, nevertheless it penetrates into plants and is deposited in their structure. Bones which contain it are slowly disaggregated by the soil, and disappear after a time under the influence of the rains.

In searching for the causes which produced these effects I have found two; the one acting rarely and feebly; the other constantly with great intensity. The first resides in a property possessed by sal ammoniac; it facilitates the solution of phosphate of lime. But although this salt dissolves a notable quantity, although it exists in small proportions in all running waters, yet this slight proportion renders its action, in this respect, inconsiderable.

The second is found in the action of carbonic acid; it is in this that the true solvent of the phoshate of lime is to be found. For, water loaded with carbonic acid dissolves large quantities of phosphate of lime, as M. Berzelius and M. Thenard had remarked. The alkalies and ebullition, by driving off or neutralising the carbonic acid, precipitate it.

The action of carbonic acid on phosphate of lime is such, that I could not doubt the effect it would produce in the bones themselves. Plates of ivory, introduced into bottles of seltzer water we softened in twenty-four hours, as if acted on by dilute hydrochloric acid. The seltzer water was loaded with phosphate of lime. So rapid is the action, so energetic, that I am sure it will be turned to some good account. It furnishes the animal matter of bones in such purity, and in a form so favorable to the manufacture of gelatine or to its direct employment as an alimentary substance, that I have no doubt it will be profitably employed in these ways.

I would call the attention of physiologists to this property. It explains the transfer of phosphate of lime into plants. It explains how bones are disaggregated and dissolved when put into the soil, under the prolonged influence of rain water containing carbonic acid. It shows how, in the animal body, bones may be dissolved by the action of venous blood, rich in carbonic acid. It explains the function of the enamel of the teeth, intended, by means of the fluorate of calcium which it contains, to protect the osseous portion against the action of the carbonic acid disengaged by the lungs and dissolved by the saliva; the latter also from being alkaline tends to counteract its injurious effects.

Would not the habitual employment of seltzer water be useful in calculi and gravel of phosphate of lime; and is it not evident that two bodies, so universally present in organic beings as carbonic acid and phoshate of lime, must react on each other under various circumstances, and give rise to phenomena of solution and precipitation extremely varied?—*South. Jour. of Med. and Phar.*

